# Pentiptycene-Derived Fluorescence Turn-Off Polymer Chemosensor for Copper(II) Cation with High Selectivity and Sensitivity

**DOI:** 10.3390/polym9040118

**Published:** 2017-03-24

**Authors:** Anting Chen, Wei Wu, Megan E. A. Fegley, Sherryllene S. Pinnock, Jetty L. Duffy-Matzner, William E. Bernier, Wayne E. Jones

**Affiliations:** 1 Department of Chemistry, Binghamton University, State University of New York, Binghamton, NY 13902, USA; achen47@binghamton.edu (A.C.); wwu39@binghamton.edu (W.W.); mfegley@binghamton.edu (M.E.A.F.); spinnoc1@binghamton.edu (S.S.P.); wbernier@binghamton.edu (W.E.B.); 2 Department of Chemistry, Augustana University, Sioux Falls, SD 57197, USA; jetty.duffy@augie.edu

**Keywords:** fluorescence, conjugated polymers, pentiptycene, chemical sensor, copper sensor

## Abstract

Fluorescent conjugated polymers (FCPs) have been explored for selective detection of metal cations with ultra-sensitivity in environmental and biological systems. Herein, a new FCP sensor, tmeda-PPpETE (poly[(pentiptycene ethynylene)-*alt*-(thienylene ethynylene)] with a *N*,*N*,*N*′-trimethylethylenediamino receptor), has been designed and synthesized via Sonogashira cross-coupling reaction with the goal of improving solid state polymer sensor development. The polymer was found to be emissive at *λ*_max_ ~ 459 nm under UV radiation with a quantum yield of 0.119 at room temperature in THF solution. By incorporating diamino receptors and pentiptycene groups into the poly[(phenylene ethynylene)-(thiophene ethynylene)] (PPETE) backbone, the polymer showed an improved turn-off response towards copper(II) cation, with more than 99% quenching in fluorescence emission. It is capable of discriminating copper(II) cation from sixteen common cations, with a detection limit of 16.5 nM (1.04 ppb).

## 1. Introduction

The development of chemical sensors has been a primary focus of environmental scientists for decades due to contamination of natural waters by toxic transition metal pollutants. Fluorescent conjugated polymers (FCPs) have received significant attention due to their ability to function as chemosensors in environmental and biological systems with both sensitivity and selectivity [1,2,3,4,5]. Based on rapid electron and energy transfer paths, these highly correlated one-dimensional systems have been found to demonstrate “million fold” sensitivity compared to monomolecular sensor analogues [6]. In most cases, the intrinsic electronic properties of the FCP backbones do not fulfill the selectivity requirements of most sensors. In response, various pendent groups known as “receptors” have been appended to the polymer side chains or at the end of the polymer chain [7]. Receptors such as crown ethers [8], amino group chelators [9], bi-pyridine and terpyridine ligands [10] and others have been evaluated based on their ability to bind metal cations selectively.

Copper is a naturally occurring trace element found in the earth’s crust and surface water. However, it can also enter water streams through industrial deposits, wood preservatives and plumbing systems. In the U.S., the Environmental Protection Agency (EPA) has a standard action level for copper of 1.3 ppm (~20 µM) [11]. Copper is also one of the important trace elements found in the living creatures. An adult human body contains about 80 mg of copper which mostly exist in various proteins and which function via redox processes [12,13]. However, excess copper may damage the cellular components and result in chronic disease, neurological disorder, and liver or kidney damage [14,15]. Copper has also been linked to Alzheimer’s, Parkinson’s and other neurodegenerative diseases [13,16]. Currently, the standard analytical method for testing copper in drinking water by the EPA is absorption spectrometry [17]. Other methods such as inductively coupled plasma mass spectroscopy (ICP-MS) [18] and electrochemical methods (cathodic or anodic stripping voltammetry) [19,20] are also used in other practice. Even though the accuracy of these instruments is high, and prized as “gold standards”, they are quite expensive and often require sample preservation and preparation, as well as expert training. In addition, these methods cannot be used in biological and clinical practice, on-site tests and in situ studies. Thus, the need of developing a simple, rapid, and adaptable method is rising in sensor research. Fluorescent chemosensors have been extensively researched due to their fast and simple read-out, high selectivity and sensitivity, and overall low price.

**Figure 1:**
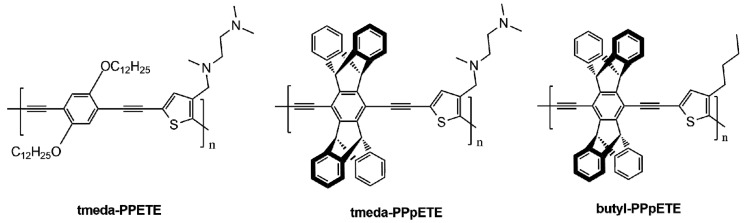
Structures of tmeda-PPETE, tmeda-PPpETE and butyl-PPpETE.

Previously, the polymer tmeda-PPETE (Figure 1), *N*,*N*,*N*′-trimethylethylene-diamino receptors loaded on the thiophene ring of the poly[(phenylene ethynylene)-*alt*-(thiophene ethynylene)] (PPETE) backbone was studied [9,21,22]. This polymer has been shown to have a small fluorescence “turn-on” response when exposed to most metal cations in solution, but a significant quenching upon addition of Cu^2+^. A more practical approach is to transfer the FCPs from solution to a solid-state matrix, and incorporate them into a field-based chemical sensing device [23,24,25]. Previous studies in the Jones group and others have shown that the photophysical properties of the solid state chemosensors are significantly affected by intermolecular interactions among polymer chains [26]. This phenomenon has been shown to significantly reduce the quantum efficiency of light emitting diodes (LEDs) and sensor devices. Thus, the transition from solution based sensor to a solid state suffers from the loss of sensitivity. In recent years, much effort has been put into chemical sensing improvements focused on minimizing the extent of polymer chain interaction to provide more efficient energy migration along the polymer chain [27,28].

In the attempt to decrease the effect of polymer self-quenching and π-stacking, a pentiptycene monomer was introduced into the polymer backbones. The rigid three-dimensional sterically hindered framework of the polymer backbones is known to prevent π-stacking of polymer chains [29]. When combined with other groups, pentiptycene can also be part of the analyte capture and recognition unit for cations and anions in small molecule sensors [30,31]. In the interest of developing cation recognition FCPs containing the pentiptycene unit, the synthesis of tmeda-PPpETE (poly[(pentiptycene ethynylene)-*alt*-(thienylene ethynylene)] with a *N*,*N*,*N*′-trimethylethylenediamino receptor) and its model polymer butyl-PPpETE are presented, together with detailed photophysical studies and sensing abilities of this new material (Figure 1).

## 2. Materials and Methods

### 2.1. Materials

Tetrakis(triphenylphosphine)palladium ((PPh_3_)_4_Pd), copper(I) iodide (CuI), tetrahydrofuran (THF), diisopropylamine (*i*-Pr_2_NH), 2,5-dibromo-3-butylthiophene and all other chemicals were purchased from Sigma-Aldrich (St. Louis, MO, USA), Fisher Scientific (Pittsburgh, PA, USA), Alfa Aesar (Ward Hill, MA, USA) or Acros Organics (Morris Plains, NJ, USA) and used as received unless otherwise noted. For air-sensitive synthetic steps, solvents were freshly distilled and stored under a nitrogen atmosphere prior to use. 

### 2.2. General Methods

NMR spectra were obtained at room temperature on a Bruker Avance III 600 spectrometer (Billerica, MA, USA). IR spectra were obtained on an FT-IR Bruker Equinox55 spectrometer at a resolution of 2 cm^−1^ with potassium bromide (KBr) pellets. The molecular weights and polydispersity distribution were determined by gel permeation chromatography (GPC) and performed by Corning Incorporated in Corning, NY, USA using THF as the mobile phase at 40 °C with a flow rate of 1.0 mL/min. The columns are calibrated using polystyrene standards ranging from 160–6,980,000 using EasiCal PS-1&2 kits. UV-Visible spectra were obtained on a Shimadzu UV-2600 UV-Visible spectrometer (Kyoto, Japan) in THF solution using 1 cm quartz cuvette cells. Fluorescence spectra were measured on a Shimadzu RF-6000 Spectro fluorophotometer. The instrument uses a 150 W Xenon arc lamp and a monochrometer with 1300 grooves per mm concave blazed holographic gratings (F/2.5). The chemosensor solutions were prepared by dissolving a specific amount of chemosensor in THF to prepare a 250 μM stock solution (with respect to the polymer repeat unit), followed by a dilution to 5 μM. All metal cation solutions were prepared by dissolving their metal chlorides in water, in order to prepare a 0.01 M stock solution, followed by a dilution into 250 μM. Metal sensing tests were conducted by titration of 250 μM metal chloride solutions via micropipette into 3 mL of the 5 μM chemosensor polymer solution in a 1 cm quartz cuvette cell followed by thorough mixing. In the selectivity test, the mole ratio of the polymer repeating unit and the metal cation were fixed at 1:1 ratio. In the titration experiment, the concentration of the polymer was fixed at 5 μM while various amount of metal cation solution were added. It was assumed that using a concentrated stock solution could minimize the influence of water on the fluorescence of the polymer. All solutions were used within 24 h from preparation. All emission experiments were repeated at least three times, and the data was very reproducible with negligible variance. The fluorescence quantum yields of all polymers were determined relative to the standard, quinine sulfate, in 0.5 M sulfuric acid (H_2_SO_4_) solution with a quantum yield of 0.546 when excited at 365 nm [32].

### 2.3. Synthesis

**Scheme 1:**
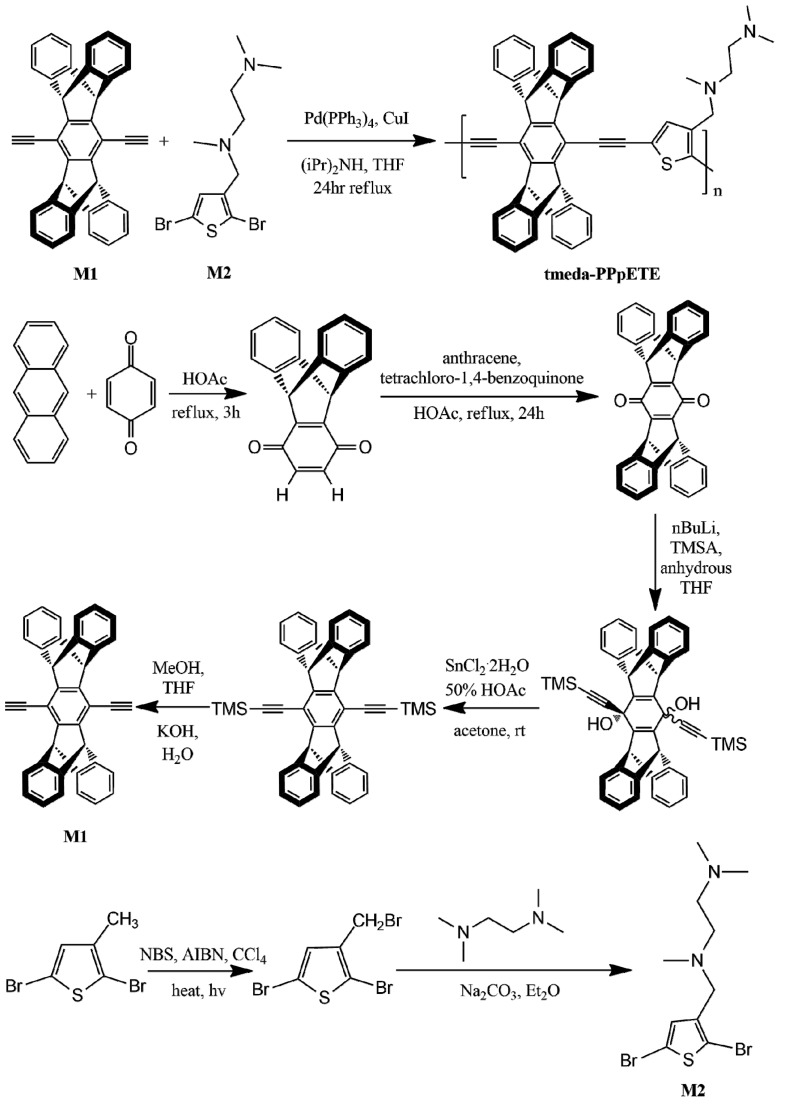
Synthesis of tmeda-PPpETE and its corresponding monomers.

The monomers, *N*-(2,5-dibromothiophene-3-ylmethyl)-*N*,*N*,*N*′-trimethylethan-1,2-diamine and 6,13-diethynyl-5,7,12,14-tetrahydro-5,14:7,12-bis([1,2-benzeno) pentacene, were prepared by modifying previous literature methods (Scheme 1) [9,33,34,35]. The polymers, tmeda-PPpETE and butyl-PPpETE were prepared by a step growth polymerization via Sonogashira reaction: a palladium catalyzed cross-coupling of aromatic halides on the thiophene ring and the terminal alkynes on the center ring of the pentiptycene group. Synthesis of butyl-PPpETE can be found in the supplementary materials.

*6,13-diethynyl-5,7,12,14-tetrahydro-5,14:7,12-bis*([1,2]*benzeno)pentacene* (**pentiptycene monomer**, **M1**) (white powder, yield 86.19%) ^1^H-NMR (600 MHz, CDCl_3_, δ_ppm_): 7.35 (q, 4H), 6.95 (q, 4H), 5.82 (s, 2H), 3.68 (s, 2H). FT-IR (ν_max_ cm^−1^, KBr): 3301, 3065, 3019, 2977, 2858, 1457, 1381, 1304, 1256, 1218, 1190, 1174, 1150, 1065, 748, 678, 661, 615, 602, 565, 449.

*N-(2,5-dibromothiophene-3-ylmethyl)-N,N,N′-trimethylethan-1,2-diamine* (**tmeda-thiophene monomer**, **M2**) (light yellow oil, yield 67.87%) ^1^H-NMR (600 MHz, CDCl_3_, δ_ppm_): 6.97 (s, 1H), 3.42 (s, 2H), 2.45 (q, 4H), 2.23 (d, 6H). FT-IR (ν_max_ cm^−1^, KBr): 3097, 2973, 2940, 2853, 2814, 2766, 2716, 1544, 1457, 1424, 1343, 1284, 1267, 1168, 1125, 1077, 1036, 1005, 981, 961, 912, 874, 836, 787, 700, 661, 580, 474.

*Poly[2,5-(3-[N-methyl-N-(N′,N′-dimethyl-2-ethanamino)methylamino] thiophenediyl)-1,2-ethynediyl-6,13-(5,7,12,14-tetrahydro-5,14,7,12-di[1,2]benzeno-pentacenediyl)-1,2-ethynediyl]* (**tmeda-PPpETE**). i-Pr_2_NH (5 mL) was added to a mixture of *N*-(2,5-dibromothiophene-3-ylmethyl)-*N*,*N*,*N*′-trimethylethan-1,2-diamine (0.374 g, 1.05 mmol), 6,13-diethynyl-5,7,12,14-tetrahydro-5,14:7,12-bis([1,2]-benzeno)-pentacene (0.479 g, 1.0 mmol), Pd(PPh_3_)_4_ (58 mg, 0.05 mmol) and CuI (20 mg, 0.10 mmol) in 30 mL anhydrous THF under nitrogen atmosphere. The resulting mixture was stirred for 5 h for the monomers to dissolve and then refluxed for 24 h. The reaction mixture was cooled down to room temperature and the residual solid in the reaction mixture was filtered out. The filtrate was concentrated under vacuum. Chloroform (30 mL × 2) was added to extract the product and the organic phase was washed with water (50 mL × 2) and dilute NaHCO_3_ solution (5 wt *%*, 50 mL × 2). The organic phase was dried over MgSO_4_, and the solvent was removed under reduced pressure. The residue was washed with hot water, hot methanol and hot acetone, sequentially. The crude product was dissolved in minimum amount of chloroform and precipitated in methanol. The precipitation product was filtered and dried to give a bright yellow solid (0.545 g, yield 81%). GPC (in THF, with polystyrene standard) $\bar{M_{w}}$ = 3.33 × 10^4^ g/mol; polydispersity index (PDI) = 2.11. ^1^H-NMR (600MHz, CDCl_3_, δ_ppm_): 7.76 (1H), 7.53 (8H), 7.07 (8H), 6.00–5.96 (4H), 4.13 (2H), 2.91–2.66 (4H), 2.39–2.34 (9H). UV-Vis *λ*_max_ = 416 nm. Emission *λ*_max_ = 459 nm. FT-IR (ν_max_ cm^−1^, KBr): 3065, 3022, 2967, 2934, 2853, 2814, 2766, 2182, 1455, 1379, 1310, 1256, 1179, 1153, 1022, 928, 853, 750, 671, 566. The formation of an internal ethynyl link was confirmed by the presence of the 2182 cm^−1^ stretch.

## 3. Results and Discussion

### 3.1. Synthesis and Characterization

**Figure 2:**
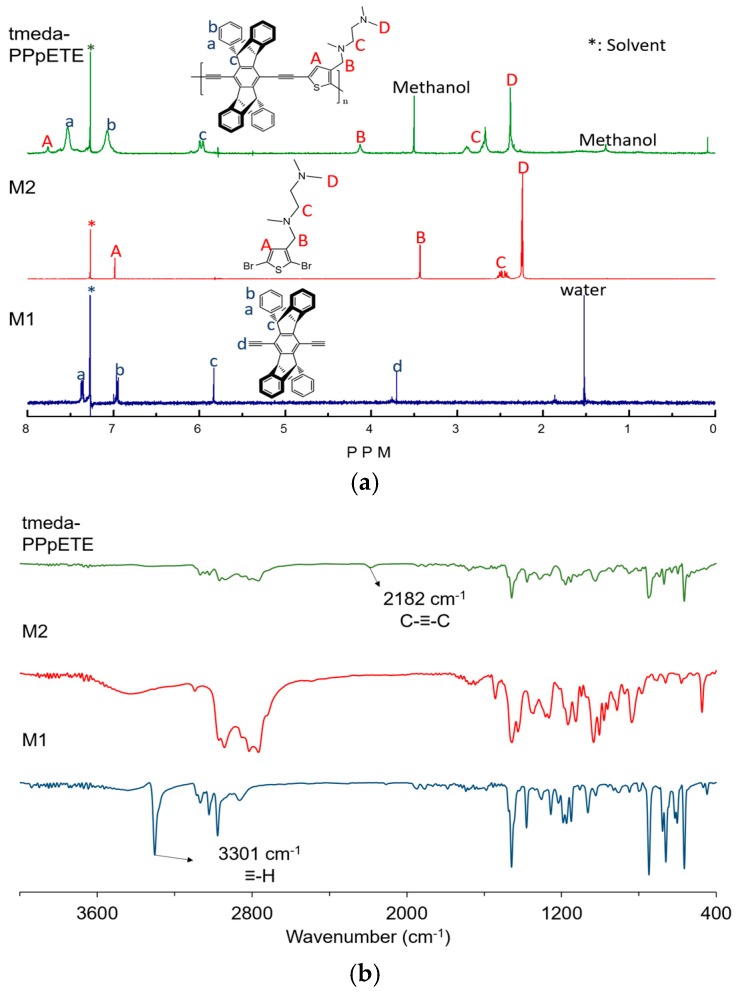
(**a**) ^1^H-NMR (600 MHz, CDCl_3_) and (**b**) FT-IR of the tmeda-PPpETE polymer and the corresponding monomers.

The polymerization of tmeda-PPpETE was carried out employing the Sonogashira cross-coupling of the aromatic halides of the receptor loaded thiophene monomer (**M2**), and the terminal alkynes on the center ring of the pentiptycene monomer (**M1**) (Scheme 1) [36]. To further study the impact of the diamino receptor, a model polymer, butyl-PPpETE was also designed and synthesized, where the diamino receptor was substituted by a butyl group [9]. **M1** and **M2** were synthesized according to literature descriptions with small adjustments (Scheme 1). All of the monomers and the polymers were well characterized by ^1^H-NMR and FT-IR (details in the Experimental Section). The NMR spectra of tmeda-PPpETE and the corresponding monomers were shown in Figure 2a. All the peaks that correspond to the protons in tmeda-PPpETE have been cleanly assigned. The success of polymerization was noted by the disappearance of the terminal alkyne peak at 3.68 ppm in the polymer NMR. It is also worth mentioning that the peak assigned to the aromatic proton on the thiophene (peak A in Figure 2a) shifted from 6.97 ppm to 7.76 ppm, and the methylene protons’ peak (peak B in Figure 2a) also shifted from 3.42 ppm to 4.13 ppm. These huge shifts can be assigned to the deshielding effect from the triple bonds [37], which also indicate the formation of internal ethynyl link. Extra evidence of polymerization is also found in the FT-IR (Figure 2b). In the pentiptycene monomer spectra, the acetylenic C-H stretching vibration has a sharp absorption at 3301 cm^−1^. This peak disappeared in the tmeda-PPpETE polymer spectra; instead, a broad, weak absorption around 2182 cm^−1^ appeared, consistent with the formation of an internal ethynyl link.

The poly [*p*-(phenyleneethynylene)-*alt*-(thinyleneethynylene)] backbone was selected because of its intense fluorescence in the visible region of the electromagnetic spectrum [10]. The tmeda diamino receptor has previously been shown to quench the PPE fluorescence by a Dexter type mechanism [22]. The pentiptycene group has a rigid three-dimensional structure, which has been widely used in conjugated polymers to prevent inter-molecular chain quenching in higher concentrations as well as in the solid state [29,38]. It is also widely accepted that this group can recognize nitrate explosives in very low concentrations in aqueous solution as well as in air [39,40]. Some computational studies suggested that this electron-rich group has two types of cavities: a large cavity near the central ring consisting of the three benzene rings, and a small cavity on the side consisting of two benzene rings. Small molecule analytes (such as nitrate explosives) are more likely to fall into the small cavity because the large cavity suffers from steric crowding [41]. In some small molecule sensors, the pentiptycene group was found to participate in recognition of cations or anions [30,31]. The newly designed FCP, tmeda-PPpETE, containing both the diamino receptor and the pentiptycene group, showed an interesting change in sensing copper(II) ions. 

The polymers tmeda-PPpETE and butyl-PPpETE are soluble in common organic solvents, such as chloroform and THF. This enables processability for application and fabrication of chemosensor systems. The molecular weight of these polymers was determined by GPC analysis with polystyrene as the standard. The molecular weight and polydispersity index were summarized in Table 1. tmeda-PPpETE has a weight average molecular weight ($\bar{M_{w}}$) of 3.33 × 10^4^ g/mol (polydispersity index (PDI) = 2.11), which corresponds to a weight average degree of polymerization $X_{w}$ ≈ 49.5 (~99 -Ph-C≡C-units). butyl-PPpETE has a slightly lower molecular weight, ($\bar{M_{w}}$) of 1.16 × 10^4^ g/mol (PDI = 2.63), which corresponds to a $X_{w}$ ≈ 18.9. The moderate conjugation and chain length was expected to provide an excellent processability of the polymer material, and, at the same time, sustain a superb “molecular wire” effect yielding signal amplification [1,8].

### 3.2. Photophysical Properties

**Figure 3:**
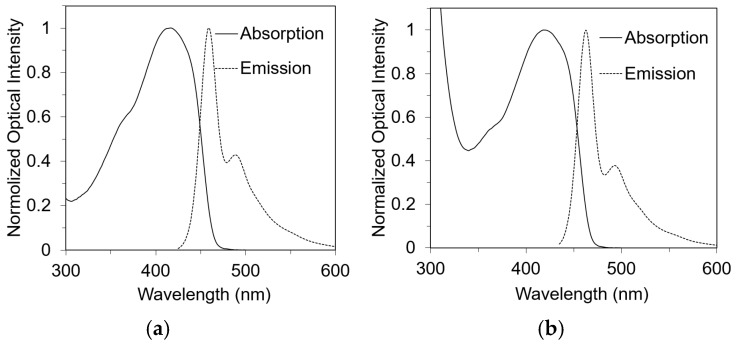
UV-Visible and emission spectra of (**a**) tmeda-PPpETE and (**b**) butyl-PPpETE in THF solution at room temperature.

The normalized UV-Visible absorption and photoluminescence emission spectra of tmeda-PPpETE and the model butyl-PPpETE are shown in Figure 3 and the photophysical data are summarized in Table 1. The polymer concentration for all photophysical experiments was maintained at 5 µM with respect to the polymer repeating unit. The polymer, tmeda-PPpETE, has an absorption from 300 nm to 480 nm and the emission maximum is around 460 nm. The polymer solutions have a light-yellow color and a strong blue emission under UV irradiation. The absorption peak can be assigned to the π-π* transition from the conjugated polymer backbone [42]. Compared to other polymers containing poly[(phenylene ethynylene)-(thiophene ethynylene)] (PPETE) backbone [9,10], the absorption peaks are blue shifted. The addition of the rigid pentiptycene group increased the π-π* transition energy, and decreased the intermolecular interaction and “self-quenching”. The photoluminescence emission of the sensor and model polymers contained a narrow peak maximum and a shoulder, which can be assigned to a single transition with vibronic structure [10]. Compared to the model polymer, butyl-PPpETE, the absorption and emission maxima of tmeda-PPpETE are both slightly blue shifted. This was also observed in previous reports on tmeda-PPETE and its model polymer [9]. The similarity in absorption and emission spectra between butyl-PPpETE and tmeda-PPpETE indicated the introduction of the diamino receptor does not lead to a significant electronic or structural distortion of the polymer backbones. The fluorescent quantum yield of tmeda-PPpETE was found to be 0.119, which is similar to other PPETE systems [9,43]. This is also sufficient to be used as a sensory material. Compared to butyl-PPpETE, which has a fluorescent quantum yield of 0.402, tmeda-PPpETE has a much lower quantum yield. The introduction of the diamino receptor results in a photo-induced electron transfer (PET) to the excited state on the polymer backbone and the resulting loss of fluorescence [9].

### 3.3. Cation Selectivity

**Figure 4:**
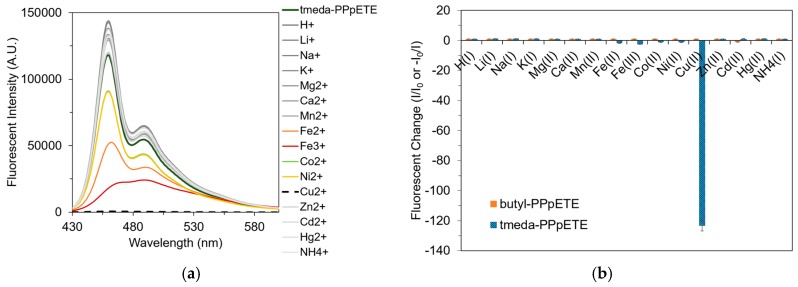
(**a**) emission spectra of tmeda-PPpETE and (**b**) the corresponding fluorescence response of tmeda-PPpETE and butyl-PPpETE to various 5 µM cations in room temperature solution. Concentration of polymers (with respect to repeat unit) were fixed at 5 µM.

Fourteen representative metal ions (Li^+^, Na^+^, K^+^, Mg^2+^, Ca^2+^, Mn^2+^, Fe^2+^, Fe^3+^, Co^2+^, Ni^2+^, Cu^2+^, Zn^2+^, Cd^2+^, Hg^2+^) as well as H^+^ and NH_4_^+^ cations were selected as representative analytes in this study. The cation titrations were carried out under the same conditions as previous studies with both the concentration of the polymer and the analyte held at 5 µM [44,45]. The average values of the fluorescent intensity (I) and the intensity change at the emission maximum upon titrating cation solutions were shown in Figure 4 (for fluorescent enhancement, the intensity change was expressed as *I*/*I*_0_; for fluorescent quenching, the intensity change was expressed as −*I*_0_/*I*, where the negative sign means the decrease in fluorescent intensity). As an electron-rich group, the pentiptycene tends to be attracted to electron withdrawing groups [29,40,41]. However, its interactions with metal cations are less well known in the literature. Yang et al. [30] presented a pentiptycene-bispyrenyl system, which was selective towards Ca^2+^ and Cd^2+^. In addition, a blue shift was observed in response to Cu^2+^, where the cation interacted with the system through a cation-π interaction. To study the mechanism of the metal cation binding, whether it is solely dependent on the diamino group or the pentiptycene group, played a role in the binding, metal cation screening was also performed on the model butyl-PPpETE polymer in the same manor.

For most cations, no significant change in fluorescence was observed; most of them showed a slight fluorescent turn-on response for both tmeda-PPpETE and the model butyl-PPpETE. However, addition of Cu^2+^ to tmeda-PPpETE caused significant fluorescent quenching, resulting more than 99% quenching at *λ*_max_ = 459 nm (Figure 4). The intensity of fluorescence at the *λ*_max_ is two magnitudes (more than 120-fold) lower compared to the initial fluorescence. The “turn-off” response of tmeda-PPpETE towards Cu^2+^ is improved from tmeda-PPETE [22]. In the case of tmeda-PPETE, only 98% of the fluorescence was quenched at saturation. The mechanism of the fluorescence quenching by Cu^2+^ was similar to tmeda-PPETE, and the enhanced performance was the result of the addition of the pentiptycene group, which prevented π-stacking and increased the quantum yield of the polymer. Titration of Fe^2+^, Fe^3+^, Co^2+^ and Ni^2+^ also decreased the fluorescence but much less significantly. It was also noticed that addition of Cd^2+^ resulted in a slight decrease of fluorescence of the model butyl-PPpETE polymer, but the same response was not observed in tmeda-PPpETE. 

**Figure 5:**
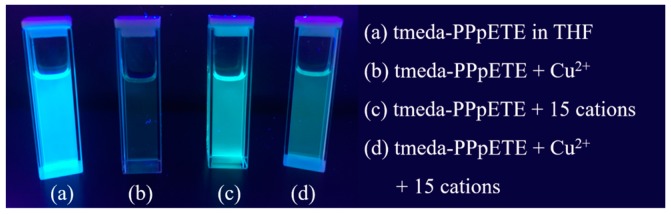
Interference studies on the tmeda-PPpETE polymer sensor: (**a**) 5 µM tmeda-PPpETE in THF; (**b**) 5 µM tmeda-PPpETE in THF after the addition of Cu^2+^ (5 µM); (**c**) 5 µM tmeda-PPpETE in THF after the addition of Li^+^, Na^+^, K^+^, Mg^2+^, Ca^2+^, Mn^2+^, Fe^2+^, Fe^3+^, Co^2+^, Ni^2+^, Zn^2+^, Cd^2+^, Hg^2+^, H^+^ and NH_4_^+^ (5 µM each); (**d**) same as (**c**) except Cu^2+^ (5 µM) was also added. The image was taken under a commercial UV lamp (excitation wavelength: 365 nm).

As shown in Figure 5, an interference study was carried out. Cuvette (a) contains the tmeda-PPpETE solution in THF, which showed an intense blue fluorescence under UV light. Cuvette (b) was the polymer solution upon addition of Cu^2+^, and the fluorescent quenching effect was visible to the naked eye. In Cuvette (c), 15 selected cations were added to the polymer solution, and the result was a slight decrease in fluorescent intensity, which was consistent with the fluorescent emission spectra. This was because Fe^2+^, Fe^3+^, Co^2+^ and Ni^2+^ can also partially quench the fluorescence, however not significantly. Cuvette (d) contained all the cations including Cu^2+^, which quenched the fluorescence significantly, demonstrating that tmeda-PPpETE can be a viable Cu^2+^ sensor. Comparing cuvette (b) and (d), it was noted that a small portion of fluorescent present when there was interference from other cations. In the previous study [46], the polymer tmeda-PPETE was preloaded with Cu^2+^, which quenches the initial fluorescence, and then a metal–polymer hybrid system can work as a turn-on sensor for iron cations. It is possible that, in the case of tmeda-PPpETE, a similar process was present where a tmeda-PPpETE/Cu^2+^ complex may be a turn-on sensor for other cations. Further studies are needed to explore the multi-ion interaction with tmeda-PPpETE. 

### 3.4. Sensitivity

**Figure 6:**
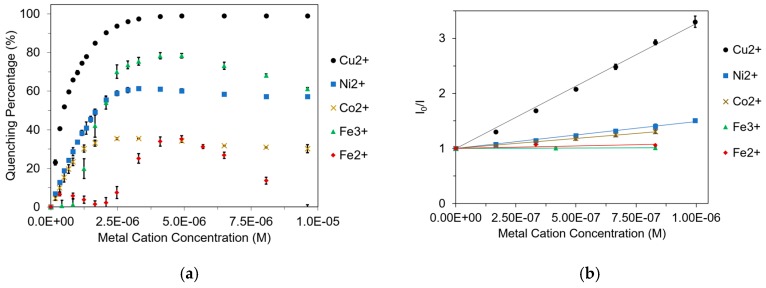
(**a**) fluorescent quenching percentage and (**b**) Sterm–Volmer plot of tmeda-PPpETE upon addition of various concentrations of Cu^2+^, Ni^2+^, Co^2+^, Fe^2+^ and Fe^3+^. The quenching percentages were calculated according to the ratio of the remainder fluorescence to the initial fluorescence $\frac{I_{0}-I}{I_{0}}\times 100\%$.

To evaluate the efficiency of tmeda-PPpETE as a Cu^2+^ sensor probe, the fluorescent emission spectra were monitored in response to a titration with various amounts of Cu^2+^ in aqueous solutions of tmeda-PPpETE (Figure 6 and Figure S4). To study the interference from other metal cations, fluorescent response of tmeda-PPpETE upon addition of Fe^2+^, Fe^3+^, Co^2+^ and Ni^2+^ was also studied (Figure 6). The fluorescence changes of tmeda-PPpETE upon adding Cu^2+^ was very dramatic and reached saturation at a very low concentration. About 80% of fluorescence was quenched at the Cu^2+^ concentration of 1.5 µM; at the concentration of 4.1 µM, more than 98.5% of initial fluorescence was quenched, and, at any concentration above that, the quenching effect was the same. Ni^2+^ and Co^2+^ showed similar titration curves where the fluorescence decrease was rapid at low concentrations and held almost the same residual fluorescence at higher concentrations. However, the quenching effect was weaker compared to Cu^2+^, Ni^2+^ could only quench 61% of the fluorescence and Co^2+^ was even less, with 35% as the maximum. Fe^2+^ and Fe^3+^ showed different titration curve shapes, where the fluorescence change was very small at lower concentration and then dramatically changed with a large margin of error in a small concentration range. After reaching saturation, the fluorescence partially recovered when more cations were added. This suggested a different mechanism of binding and quenching event compared to Cu^2+^, Ni^2+^ and Co^2+^. Further investigation is needed to explore the binding with Fe^2+^ and Fe^3+^ and the difference between them; however, this paper will focus only on the selectivity and sensitivity of Cu^2+^. Considering all the interfering ions, only Cu^2+^ will cause fluorescence quenching more than 79%. Tracing on the curve, it was noted that the concentration of Cu^2+^ was 1.37 µM (87.4 parts per billion, ppb) when the quenching percentage reaches 79%. This is still much lower than the EPA action level of Cu^2+^ in water which is 1.3 ppm, which means that tmeda-PPpETE can be a viable sensor for Cu^2+^ screening.

. 

To further quantify the quenching efficiency of the tmeda-PPpETE, the fluorescence intensities of the polymer and the concentration of various metal ions were fitted into the Stern–Volmer equation:

(1) $$\frac{I_{0}}{I}=1+K_{\mathrm{SV}}\;[M],$$

where *I*_0_ and *I* are the fluorescence intensity of the polymer before and after the addition of metal ions; and *K*_sv_ is the Stern–Volmer quenching constant. The results and linear regression are shown in Figure 6b and the values are tabulated in Table 2.

The *K*_sv_ value of Cu^2+^ is an order of magnitude larger than the rest of the ions, indicating good selectivity at low concentrations. The limit of detection (LoD) for Cu^2+^ was determined by the following equation:

(2) $$\mathrm{LoD}=\frac{3\times \sigma }{s},$$

where σ is the standard deviation of the blank signals; and s is the slope of the calibration curve. The LoD of Cu^2+^ was determined to be 1.65 × 10^−8^ M or 1.04 ppb (µg/L). The high sensitivity of tmeda-PPpETE is capable of screening trace copper under the EPA standard (1.3 ppm).

## 4. Conclusions

A new FCP, tmeda-PPpETE, with the PPETE conjugated backbone, diamino receptors and pentiptycene groups was designed and synthesized via the Sonogashira cross coupling reaction. Screening of sixteen common cations showed that this sensor was selective to copper(II) cations with a fluorescent quenching response over 120-fold at *λ*_max_ = 490 nm. At lower concentrations, the fluorescent quenching and the concentration of Cu^2+^ has a linear relationship, with a Stern–Volmer quenching constant *K*_sv_ of 2.27 × 10^6^, which is significantly larger than for any other cations. The detection limit of tmeda-PPpETE to Cu^2+^ is 1.04 ppb, which is much lower than the EPA action level of 1.3 ppm. This is the first demonstration of the pentiptycene group participating in metal cation interaction in a polymer system.

## Figures and Tables

**Table 1:** Summary of molecular weight and photophysical properties of polymers.

Polymer Name	$\bar{M_{w}}$ (g/mol)	$\bar{M_{n}}$ (g/mol)	PDI	Absorption *λ*_max_ (nm)	Emission *λ*_max_ (nm)	Fluorescence Quantum Yield
tmeda-PPpETE	3.33 × 10^4^	1.58 × 10^4^	2.11	416	459	0.119
butyl-PPpETE	1.16 × 10^4^	4.43 × 10^3^	2.63	418	463	0.402

**Table 2:** Summary of linear regression equations, *R*^2^ values and *K*_sv_ values for various cations from Stern–Volmer graph.

Cation Name	Linear Regression Equation	*R*^2^ Value	*K*_sv_
Cu^2+^	I0I=1+2.27×106[Cu2+]	1.00	$2.27\times 10^{6}$
Ni^2+^	I0I=1+4.90×105[Ni2+]	1.00	$4.90\times 10^{5}$
Co^2+^	I0I=1+3.63×105[Co2+]	1.00	$3.63\times 10^{5}$
Fe^3+^	I0I=1+1.65×104[Fe3+]	1.00	$1.65\times 10^{4}$
Fe^2+^	I0I=1+9.32×104[Fe2+]	0.39	$9.32\times 10^{4}$

## Supplementary Materials

The following are available online at www.mdpi.com/2073-4360/9/4/118/s1, Synthesis of M1 and butyl-PPpETE, Figure S1: 1H-NMR of the monomers and the butyl-PPpETE polymer (600 MHz, CDCl3); Figure S2: FT-IR of butyl-PPpETE and its corresponding monomers; Figure S3: Emission spectra of the interference studies on the tmeda-PPpETE polymer sensor; Figure S4: Fluorescence emission spectra of tmeda-PPpETE upon addition of different concentrations of Cu^2+^.
