# Supplementary material for: Pentiptycene-Derived Fluorescence Turn-Off Polymer Chemosensor for Copper(II) Cation with High Selectivity and Sensitivity
Source: Polymers (Basel). 2017 Mar 24;9(4):118. doi: 10.3390/polym9040118 (PMC6432232; doi:10.3390/polym9040118)
Supplement: Supplementary file 1 [file polymers-09-00118-s001.pdf]

Supplementary Materials: Pentiptycene-Derived Fluorescence Turn-Off Polymer Chemosensor for Copper(II) Cation with High Selectivity and Sensitivity

Anting Chen, Wei Wu, Megan E. A. Fegley, Sherryllene S. Pinnock, Jetty L. Duffy-Matzner, William E. Bernier and Wayne E. Jones Jr

1. **Synthesis of M1**

9,10-Dihydro-9,10-o-benzenoanthracene-1,4-dione

A mixture of anthracene (20.72 mmol, 3.6926 g) and 1,4-benzoquinone (102.4 mmol, 11.0654 g) in 150 mL of acetic acid was refluxed for 3 hours. The reaction mixture was then poured into cold water and the precipitate was filtered. The brown crude product was then washed with hot water and purified by column chromatography using chloroform as the mobile phase. The product is a yellow powder (4.7630 g, yield 80.86%). ^{1}H NMR (600 MHz, $CDCl_3$, δ_{ppm}): 7.44 (q, 4H), 7.05 (q, 4H), 6.61(s, 2H), 5.81(s, 2H).

5,6,7,12,13,14-Hexahydro-5,14:7,12-bis(o-benzeno)-pentacene-6,13-dione

A mixture of 9,10-Dihydro-9,10-*o*-benzenoanthracene-1,4-dione (16.75 mmol, 4.7630 g), anthracene (16.78 mmol, 2.9913 g) and tetrachloro-1,4-benzoquione (17.04 mmol, 4.1894 g) in 300 mL of acetic acid was refluxed for 24 hours. After the reaction was cooled to room temperature, the solid was filtered and washed with diethyl ether, and then dried under vacuum to yield a yellow solid (6.9416 g, yield 89.99%). ^{1}H NMR (600 MHz, $CDCl_3$, δ_{ppm}): 7.36 (q, 8H), 6.98 (q, 8H), 5.76 (s, 4H).

(5,6,14)-6,13-bis((trimethylsilyl)ethynyl)-5,6,7,12,13,14-hexahydro-5,14:7,12-bis([1,2]benzeno)pentacene-6,13-diol.

Under nitrogen protection, n-butyllithium (2.5 M in Hexanes, 37.68 mmol, 15.07 mL) was added dropwise to a solution of (trimethylsilyl)acetylene (37.68 mmol, 3.7009 g) in 50 mL of THF in salt ice bath. The mixture was kept stirring at 0 °C for another 40 min before it was transferred to a solution of 5,6,7,12,13,14-Hexahydro-5,14:7,12-bis(*o*-benzeno)-pentacene-6,13-dione (15.50 mmol, 6.9334 g) in 100mL of THF. The reaction mixture was warmed to room temperature and stirred overnight. The reaction was quenched by adding 5 mL of saturated ammonium chloride solution. The reaction mixture was poured into a separatory funnel and chloroform was added to extract the product. The organic layer was washed with water and dried over magnesium sulfate. After the solvent was removed under reduced pressure, hexane was added to the residue and product was collected by vacuum filtration to yield white solid (9.9035 g, yield 100%). ^{1}H NMR (600 MHz, $CDCl_3$, δ_{ppm}): 7.32 (m, 8H), 6.92 (m, 8H), 5.5 (s, 4H), 0.19 (s, 18H).

(5,14)-6,13-bis((trimethylsilyl)ethynyl)-5,7,12,14-tetrahydro-5,14:7,12-bis([1,2]benzeno)pentacene

The crude product from the last step (15.07 mmol, 9.9035 g) was dissolved in 120 mL of acetone. A solution of tin(II) chloride dihydrate ($SnCl_2{\cdot}2H_2O$, 39.30 mmol, 8.8680 g) in 120 mL of 50% acetic acid was added to the acetone solution dropwise under nitrogen protection. The mixture was stirred at room temperature for 24 hours before the product was filtered. The solid was then dissolved in chloroform, washed with water and 5% sodium bicarbonate and dried over magnesium sulfate. The solid was filtered and solvent was removed under reduced pressure and the residue was washed with hexane to remove the yellow impurities. The resulting white solid was collected by vacuum filtration (7.8077 g, yield 83.15%). ^{1}H NMR (600 MHz, $CDCl_3$, δ_{ppm}): 7.34 (q, 8H), 6.96(q, 8H), 5.79 (s, 4H), 0.50 (s, 18H).

6,13-diethynyl-5,7,12,14-tetrahydro-5,14:7,12-bis([1,2]benzeno)pentacene (***pentiptycene monomer, M1***)

The deprotection of the trimethylsilyl group was carried out by dissolving (5,14)-6,13-bis((trimethylsilyl)ethynyl)-5,7,12,14-tetrahydro-5,14:7,12-bis([1,2]benzeno)pentacene in a mixture solvent (25% methanol, 25% concentrated potassium hydroxide (20 wt %) and 50% THF). The cloudy mixture was stirring under room temperature for 6hrs. The product was collected by vacuum filtration after the stirring. (white powder, yield 86.19%) ^{1}H NMR (600 MHz, $CDCl_3$, δ_{ppm}): 7.35 (q, 4H), 6.95 (q, 4H), 5.82 (s, 2H), 3.68 (s, 2H). FT-IR (ν_{max}, cm^{-1}, KBr):3301, 3065, 3019, 2977, 2858, 1457, 1381, 1304, 1256, 1218, 1190, 1174, 1150, 1065, 748, 678, 661, 615, 602, 565, 449.

2. Synthesis of the Model Polymer, Butyl-PPpETE

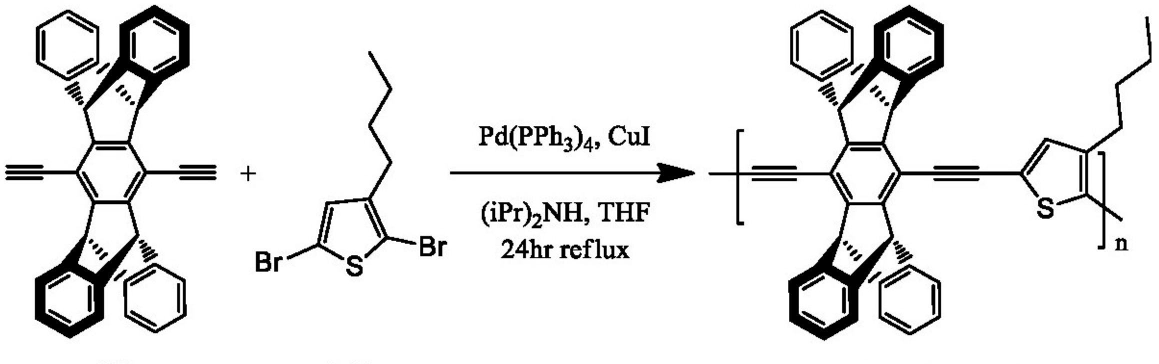

Scheme S1 Synthesis of butyl-PPpETE.

Poly[2,5-(3-butylthiophenediyl)-1,2-ethynediyl-6,13-(5,7,12,14-tetrahydro-5,14,7,12-di[1,2]benzeno-pentacenediyl)-1,2-ethynediyl] (***butyl-PPpETE***)

i-Pr_2NH (10 mL) was added to a mixture of 2,5-dibromo-3-butylthiophene (0.5952 g, 2.0 mmol), 6,13-diethynyl-5,7,12,14-tetrahydro-5,14:7,12-bis([1,2]benzeno)pentacene (0.9553 g, 2.0 mmol), $Pd(PPh_3)_4$ (0.1156 g, 0.10 mmol) and CuI (0.0381 g, 0.20 mmol) in 50 mL anhydrous THF under nitrogen atmosphere. The resulting mixture was stirred for 5 hours for the monomer to dissolve and then refluxed for 24 hours. The reaction mixture was cooled down to room temperature and residual solid in the reaction mixture was filtered out before solvent was removed under vacuum. Chloroform (30 mL × 2) was added to extract the product and the organic phase was washed with water (50 mL × 2) and dilute $NaHCO_3$ solution (5 wt %, 50 mL × 2), sequentially. The organic phase was dried over $MgSO_4$, and the solvent was removed under reduced pressure. The residue was washed with hot water, hot methanol and hot acetone. The crude product was dissolved in minimum amount of chloroform and precipitated in methanol and gave a bright yellow solid (1.185 g, yield 96%). GPC (in THF, with polystyrene standard) average molecular weight 1.16×10^4 g/mol; polydispersity index (PDI) = 2.63. ^{1}H NMR (600MHz, $CDCl_3$, δ_{ppm}): 7.64 (1H), 7.53 (8H), 7.08 (8H), 5.99-5.96 (4H), 3.24 (2H), 2.10 (2H), 1.78 (2H), 1.28-1.18 (3H). UV-Vis λ_{max} = 418 nm. Emission λ_{max} = 463 nm. FT-IR (ν_{max} cm^{-1}, KBr): 3067, 3018, 2955, 2925, 2855, 2180, 1458, 1379, 1311, 1177, 1152, 1101, 1021, 933, 843, 750, 671, 565. The formation of an internal ethynyl link was confirmed by the presence of the 2180 cm^{-1} stretch.

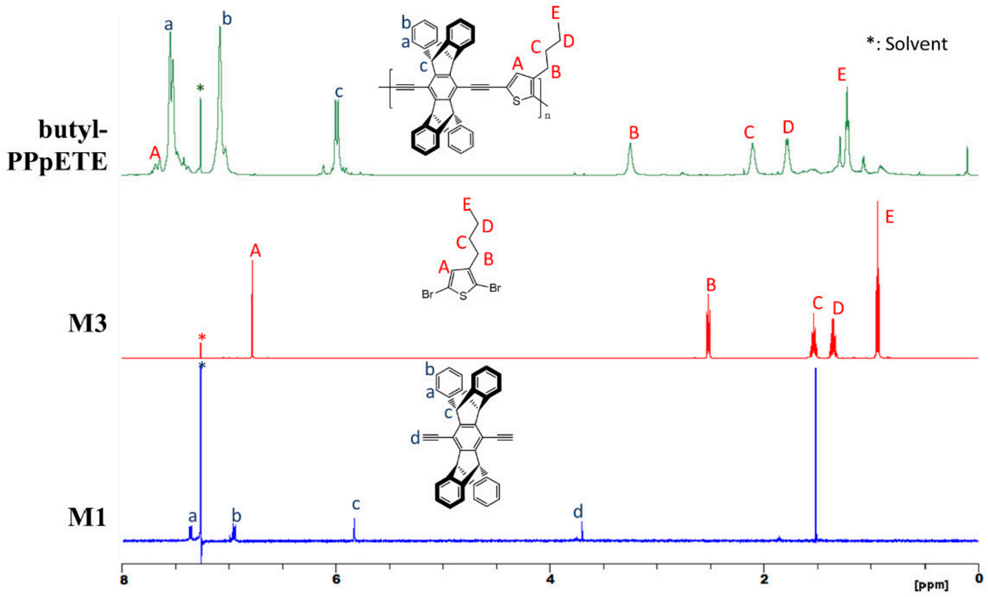

Figure S1. ^{1}H-NMR of the monomers and the butyl-PPpETE polymer (600 MHz, $CDCl_3$).

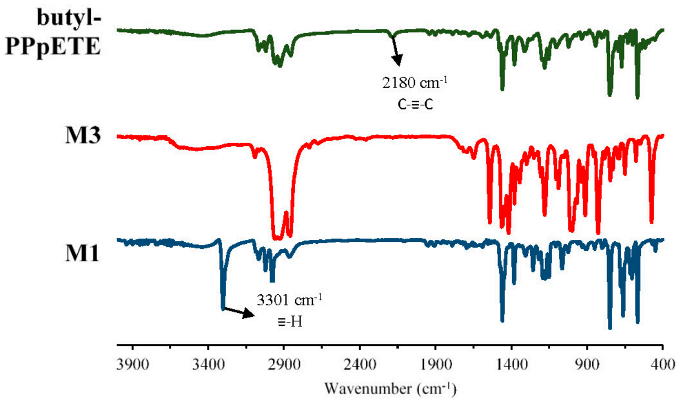

Figure S2. FT-IR of butyl-PPpETE and its corresponding monomers.

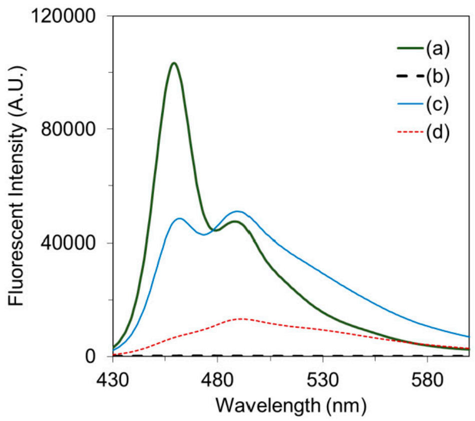

Figure S3. Emission spectra of the interference studies on the tmeda-PPpETE polymer sensor: (a) 5 μM tmeda-PPpETE in THF; (b) 5 μM tmeda-PPpETE in THF after the addition of Cu^{2+} (5 μM); (c) 5 μM tmeda-PPpETE in THF after the addition of Li^{+}, Na^{+}, K^{+}, Mg^{2+}, Ca^{2+}, Mn^{2+}, Fe^{2+}, Fe^{3+}, Co^{2+}, Ni^{2+}, Zn^{2+}, Cd^{2+}, Hg^{2+}, H^{+} and NH^{4+} (5 μM each); (d) Same as (c) except Cu^{2+} (5 μM) was also added.

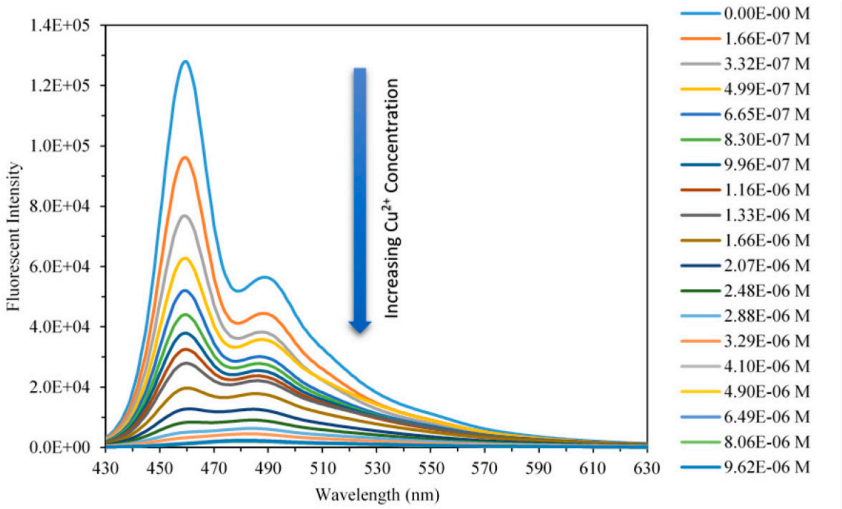

Figure S4. Fluorescence emission spectra of tmeda-PPpETE upon addition of different concentrations of Cu^{2+}.
